# Optimization of Oxygen Evolution Reaction with Electroless Deposited Ni–P Catalytic Nanocoating

**DOI:** 10.3390/nano11113010

**Published:** 2021-11-09

**Authors:** Sergio Battiato, Mario Urso, Salvatore Cosentino, Anna Lucia Pellegrino, Salvo Mirabella, Antonio Terrasi

**Affiliations:** 1 IMM-CNR, Università di Catania, Via S. Sofia 64, I-95123 Catania, Italy; mario.urso@ct.infn.it (M.U.); salvatore.cosentino@ct.infn.it (S.C.); salvo.mirabella@dfa.unict.it (S.M.); antonio.terrasi@ct.infn.it (A.T.); 2 Dipartimento di Scienze Chimiche, Università degli Studi di Catania, INSTM UdR Catania, Viale Andrea Doria 6, I-95125 Catania, Italy; annalucia.pellegrino@unict.it

**Keywords:** oxygen evolution reaction, nickel phosphide, electrocatalysis, electroless deposition, catalytic nanocoatings

## Abstract

The low efficiency of water electrolysis mostly arises from the thermodynamic uphill oxygen evolution reaction. The efficiency can be greatly improved by rationally designing low-cost and efficient oxygen evolution anode materials. Herein, we report the synthesis of Ni–P alloys adopting a facile electroless plating method under mild conditions on nickel substrates. The relationship between the Ni–P properties and catalytic activity allowed us to define the best conditions for the electroless synthesis of highperformance Ni–P catalysts. Indeed, the electrochemical investigations indicated an increased catalytic response by reducing the thickness and Ni/P ratio in the alloy. Furthermore, the Ni–P catalysts with optimized size and composition deposited on Ni foam exposed more active sites for the oxygen evolution reaction, yielding a current density of 10 mA cm^−2^ at an overpotential as low as 335 mV, exhibiting charge transfer resistances of only a few ohms and a remarkable turnover frequency (TOF) value of 0.62 s^−1^ at 350 mV. The present study provides an advancement in the control of the electroless synthetic approach for the design and large-scale application of high-performance metal phosphide catalysts for electrochemical water splitting.

## 1. Introduction

Electrochemical water splitting has aroused unremitting attention as a promising method to produce H_2_, a clean and sustainable energy carrier considered an alternative to fossil fuels [1,2,3]. Despite its great potential, electrolytic water generation is a thermodynamically uphill process, since the two semi-reactions involved, the hydrogen evolution reaction (HER) and the oxygen evolution reaction (OER), are constrained by sluggish kinetics, particularly the OER, which undergoes a complex four-electron transfer process [4,5]. At present, the main strategy to enhance the efficiency of the OER half-reaction involves the use of noble-metal-based (e.g., RuO_2_ and IrO_2_) electrocatalysts, on account of their superior catalytic performance [6]. Nonetheless, the large-scale application of these materials is hampered by their low abundance and high cost [7,8,9]. Consequently, it is mandatory to seek earth-abundant and efficient OER electrocatalysts [10]. In this context, transition metal-based catalysts are undoubtedly compelling candidates in view of their abundancy, cost effectiveness and great potential in the field of OER for electrolytic water splitting [11,12]. Among the multitude of transition metals which have been explored as OER electrocatalysts, Ni-based catalysts have been widely demonstrated to have noteworthy catalytic properties [13,14,15,16]. Several research studies have proved that alloying Ni with P provides a beneficial effect for the OER process [17,18,19,20]. The improved catalytic activity of transition metal phosphide catalysts arises from the partially charged nature of the Ni (δ^+^) and P (δ^−^) atoms in the Ni–P alloy. In particular, Ni δ^+^ and P δ^−^ promote the formation of more catalytically active NiOOH and phosphate species at the near surface of the catalysts that act as the active phases during the OER, thus improving the catalytic activities [21,22].

A plethora of methods have been proposed to prepare nickel phosphide catalytic materials including straightforward hydrothermal [23], electrodeposition [24,25] and electroless deposition [26] techniques.

Although the above methods have been successful in the development of nickel phosphides with a discrete OER activity, the efficiency of these materials is not yet in step with that of the noble metals. In our opinion, a decisive enhancement of the catalytic efficiency of these catalysts may be obtained by a careful design of the electrocatalyst characteristics in terms of size, shape and composition. Favorably improving these properties can be achieved through proper control of the synthetic process employed for the realization of the Ni–P catalysts. In addition, the high catalyst loading of these materials that is often reported in the abovementioned synthetic methods affects the mass transport of reactants and ions due to the thicker catalyst layers. An effective strategy to enhance the inherent activities of electrocatalysts is the realization of ultra-small sized catalysts since the exposed active sites can offer large specific surface areas for improving the electron and mass transfer.

Triggered by these considerations, we dedicated ourselves to comprehensively investigating the electroless synthesis of Ni–P catalysts on nickel substrates, whose fine control led to the realization of few-nanometer-sized amorphous Ni–P alloys with tunable Ni/P ratios. An in-depth understanding encompassing the synthesis parameters—film properties and film property–OER performance relationships—was achieved. Electrochemical measurements showed that the highest OER performance was attained in the thinnest Ni–P nanocoatings. Tuning the Ni–P composition plays a crucial role in governing the electrocatalytic activity of the alloy, as demonstrated by the increased performance of the Ni–P alloys with the highest phosphorus content. Moreover, the samples deposited on nickel foam showed prominently enhanced OER activity, needing an overpotential of only 335 mV to sustain a current density of 10 mA cm^−2^, showing low Tafel slope (70 mV dec^−1^) and charge transfer resistance (4 ohms) values. Noticeably, the activity of the present Ni–P alloy is amid the best performances of transition metal phosphides.

Furthermore, the present low-cost and controllable synthetic approach can be easily extended to fabricate other ultra-small metal phosphide materials with tailored metal–phosphorus compositions, providing a significant platform for the future development of high-efficiency electrocatalysts.

## 2. Materials and Methods

### 2.1. Substrates

The substrates used for this study consisted of a 100 nm nickel layer deposited by evaporation on silicon substrates (hereafter Ni flat), and nickel foams (Goodfellow Inc., Huntingdon, England, thickness 1.6 mm, porosity ≥95%) purposely chosen to realize an electrode with high porosity. Prior to synthesis, both substrates were carefully cleaned with acetone, isopropanol and deionized (DI) water and dried in N_2_.

### 2.2. Preparation of Ni–P Catalysts

The electroless Ni–P alloys were synthesized in a solution containing 25 g L^−1^ nickel sulfate hexahydrate, which serves as the nickel source, 20 g L^−1^ sodium citrate as a complexing agent and 30 g L^−1^ ammonium fluoride as a stabilizer. Sodium hypophosphite, which acts as a reducing agent, was added into the bath in different concentrations in order to optimize the compositions of the Ni–P alloys, while the concentrations of all other reagents were kept constant. The corresponding alloy coatings are hereafter referred to as Ni–P_17_, Ni–P_21_ and Ni–P_24_ on the basis of the P percentage in the alloy (as calculated from Rutherford backscattering spectrometry) corresponding to the addition of 7.5, 15 and 30 g L^−1^ sodium hypophosphite in the bath (see Table 1).

The pH of the solution was measured to be 5.05, the plating bath was heated to 70 °C and the thickness of the coatings was controlled by varying the deposition time (3, 4 and 6 min).

### 2.3. Characterization

Film structure was analyzed by X-ray diffraction using a Smartlab Rigaku diffractometer (Rigaku Corporation, Tokyo, Japan) in grazing incidence mode (0.5°), equipped with a rotating anode of Cu K_α_ radiation operating at 45 kV and 200 mA. The surface morphology was characterized using a scanning electron microscope (Gemini field emission SEM Carl Zeiss SUPRA 25, Carl Zeiss AG, Oberkochen, Germany). Rutherford backscattering spectrometry (RBS) measurements were carried out using a 2.0 MeV He^+^ ion beam that hits the sample surface at normal incidence, while backscattered He^+^ atoms were detected at an angle of 15° out of the normal to the surface. The Ni–P dose in the Ni–P alloys was determined by RBS spectra simulation using SIMNRA software [27]. Once the Ni–P dose is known, the layer thickness is derived by dividing the Ni–P dose in at/cm^2^ for the atomic density, expressed in at/cm^3^, which is calculated as follows:

$$\mathrm{Ni}–P\;\mathrm{atomic}\;\mathrm{density}\;\left(\frac{\mathrm{at}}{{\mathrm{cm}}^{3}}\right)=\frac{\mathrm{Ni}–P\;\mathrm{mass}\;\mathrm{density}\;\left(\frac{g}{{\mathrm{cm}}^{3}}\right)}{\mathrm{Ni}–P\;\mathrm{molecular}\;\mathrm{weight}\;\left(\frac{g}{\mathrm{mol}}\right)}{\;\cdot \;\mathrm{Avogadro}}^{’}s\;\mathrm{number}\left(\frac{\mathrm{at}}{\mathrm{mol}}\right)$$

The Ni–P mass density depends on the Ni–P composition [28]; thus, the resulting atomic densities were 8.48·10^22^ Ni–P/cm^3^, 8.65·10^22^ Ni–P/cm^3^ and 8.79·10^22^ Ni–P/cm^3^ for the Ni–P_17,_ Ni–P_21_ and Ni–P_24_ samples, respectively.

### 2.4. Electrochemical Tests

Catalytic performances of the Ni–P catalysts were evaluated using a Versastat-4 potentiostat in a three-electrode setup with a Pt wire as the cathode, a saturated calomel electrode (SCE) as the reference electrode and Ni–P catalysts on Ni flat and Ni foam as the working electrodes. The current density was normalized to the geometrical surface area, and the measured potentials vs. Hg/Hg_2_Cl_2_ were converted to the reversible hydrogen electrode (RHE) according to the equation [29,30]

E_RHE_ = E_Hg_/_Hg2Cl2_ + 0.059 pH + 0.241

All samples were activated acquiring ten cyclic voltammetry (CV) cycles at 10 mV s^−1^ until the curves were stable [31,32], and all potentials measured were manually corrected by iR_u_ compensation as follows:

η = η’−iR_u_

where i is the current collected at the electrode, while R_u_ [ohm] is the intercept on the real axis of the Nyquist plots (real vs. imaginary part of the impedance, Z) obtained from the electrochemical impedance spectroscopy (EIS) measurements. Steady-state Tafel plots were acquired from chronopotentiometric (CP) measurements in steps from 0.1 to 10 mA cm^−2^, each held for 3 min.

EIS was performed in potentiostatic mode from 100 to 0.1 kHz, with an AC voltage of 5 mV. All measurements were performed at room temperature and atmospheric pressure, in a one-compartment electrochemical Teflon cell filled with 1 M KOH as the supporting electrolyte.

## 3. Results and Discussion

Ni–P alloys with various percentages of P were synthesized using a facile electroless deposition approach.

The formation process is driven by an autocatalytic reduction reaction on the surface of the Ni substrate. Ni is reduced by capturing the electrons provided by the reducing agent (NaH_2_PO_2_), while zero-valence P and H_2_ are formed as by-products.

The chemical reaction can be described as follows [33]:

Ni^2+^ + H_2_PO^−^_2_ + H_2_O → Ni + HPO_3_^2−^ + 3H^+^

3H_2_PO^−^_2_ → H_2_PO^−^_3_ +H_2_O + 2OH^−^ + 2P (1)

H_2_PO^−^_2_ + H_2_O → H_2_PO^−^_3_ +H_2_

**Figure 1:**
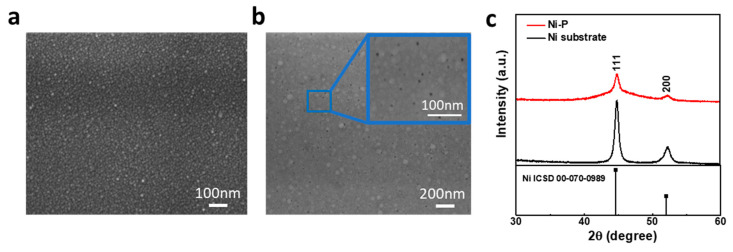
SEM images of (**a**) Ni substrate and (**b**) Ni–P_24_ deposited for 6 min. (**c**) X-ray diffraction patterns of electroless-deposited Ni–P (red pattern) and Ni substrate (black pattern).

Scanning electron microscopic analysis was performed to understand the morphology and size of the product. Figure 1a shows the SEM image of the Ni substrate, presenting a homogeneous smooth surface. Figure 1b shows the micrography of a Ni–P_24_ film deposited on the same Ni substrate for 6 min, which consists of a homogeneous layer with a grain size below 10 nm. Interestingly, the higher-magnification image (inset figure) depicts the presence of pinholes scattered on the surface. These pinholes can be reasonably ascribed to the hydrogen evolution taking place during the plating process, as reported by other authors [33,34]. SEM analysis of a film after the OER is useful in evaluating how the surface of the catalyst is affected by the electrochemical activity; in this regard, the literature often reports major modifications of the morphology after the OER, indicating significant changes in the surface chemistry [35].

In our case, the morphology of the same Ni–P_24_ film after the oxygen evolution reaction (OER) was retained compared to the pristine one, as shown in Figure S1.

The phase purity of the synthesized materials was revealed using X-ray diffraction (XRD). The pattern of the nickel substrate was acquired as well and compared to that of the Ni–P films. It is worth noting that the grazing incidence acquisition mode allows us to detect only the extreme surface of the sample, thus avoiding the contribution of the underlying nickel substrate. In Figure 1c, the X-ray diffraction (XRD) pattern obtained for the nickel substrate (black pattern) shows characteristic peaks centered at 2θ = 44.6° and 52.1° matching the reflections of the (111) and (200) lattice planes typical of the face-centered cubic (FCC) nickel phase (ICSD diffraction card 00-070-0989), which points to a crystalline nature of the substrate. Concerning the pattern obtained for the Ni–P_24_ sample (red pattern), in this case, a diffuse scattering peak is clearly observable around 44.6°. The widening of this peak, commonly observed in electroless-deposited Ni–P alloys, is a hint that P has entered the face-centered cubic nickel lattice and clearly indicates that the as-deposited Ni–P is amorphous [17,36,37,38].

**Figure 2:**
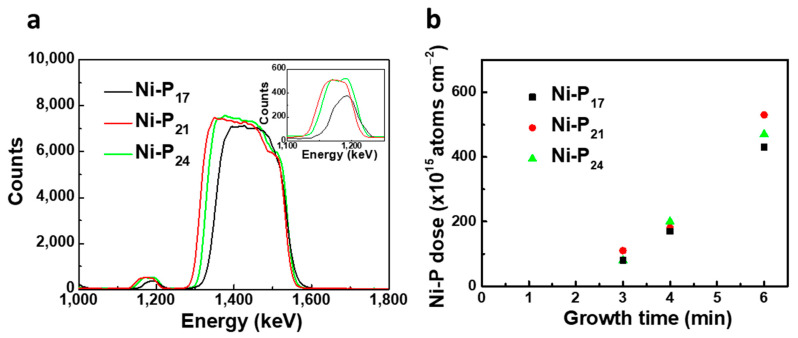
(**a**) Comparison of RBS spectra of Ni–P coatings with different phosphorus concentrations grown for 6 min. The inset reports a zoom in on the region of phosphorus. (**b**) Plot of the dose of Ni–P as a function of the growth time.

Figure 2 shows the RBS spectra of our Ni–P samples. In Figure 2a, we report the RBS spectra of the Ni–P coatings grown for 6 min at different hypophosphite concentrations. For all samples, we observed the presence of the following peaks: the peak at 1.17 MeV is related to He^+^ backscattered by P, whereas the band diffused in the range between 1.32 and 1.52 MeV arises from the He^+^ backscattered by Ni of the substrate (lower energy) and Ni of the Ni–P film (higher energy). The height of each RBS signal depends on the atomic density and atomic number of the target. Moreover, an increase in the P signals with the increasing hypophosphite concentration was observed (the reader can compare the zoomed P regions of RBS spectra, shown in the inset of Figure 2a). The quantitative RBS analysis of the same spectra carried out by SIMNRA simulation allowed us to calculate the Ni/P ratio in the alloys. The reason why the films’ composition was determined at the longest deposition time of 6 min is that the calculation performed on the thickest samples is more reliable. Indeed, a physiological margin of error occurred in the simulation of the spectra of thinner samples (the sample deposited for 3 min had a thickness of only about 7 nm), the substrate being constituted by the same Ni element. Concerning the synthesis characteristics, some research studies have described an incubation period before the beginning of Ni–P plating [39,40]. Here, we extrapolated the incubation time by fitting the curves of the Ni–P dose vs. the growth time, as reported in Figure 2b. Notably, the incubation time values found in the three samples are substantially superimposable, with a mean value of about 2.4 min, indicating that the hypophosphite concentration does not impact this aspect of the synthesis.

**Figure 3:**
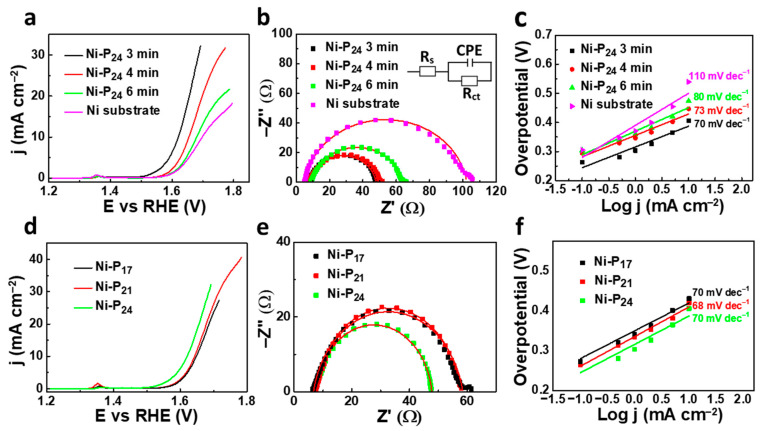
(**a**) Polarization curves, (**b**) Nyquist plots with the fitting curves plotted and (**c**) Tafel plots of Ni–P_24_ coatings deposited at different growth times and the Ni substrate; (**d**) polarization curves, (**e**) Nyquist plots and (**f**) Tafel plots of the Ni–P coatings with different percentages of phosphorus deposited for 3 min.

To reveal the OER activities of the as-prepared samples, various electrochemical measurements of Ni–P samples were carried out in alkaline media. In Figure 3a, we report a portion of the 10th CV cycle of Ni–P_24_ samples deposited at different times in the region between 1.2 and 1.8 V, where the typical charge transfer reactions occur. Firstly, a redox peak at ∼1.38 V (vs. RHE) is visible in the polarization plot, which can be safely attributed to the formation of surface NiOOH species, consistent with previous reports of other nickel-based electrocatalysts [41,42]. The comparison of the curves indicated that the Ni–P_24_ sample deposited for 3 min exhibited an improved OER performance, as evidenced by the overpotential of 388 mV required to attain a current density of 10 mA cm^−2^, which is lower compared to the values obtained for Ni–P_21_ deposited at longer times. In comparison, the bare nickel electrode showed poor electrochemical activity, evidencing the beneficial role of phosphorus in the catalytic activity. To further elucidate the electrode kinetics, electrochemical impedance spectroscopy (EIS) was performed. The experimental EIS spectra, shown in Figure 3b, were fitted by a simplified Randles circuit (inset of the same figure). Nyquist plots were acquired at a fixed potential of 1.6 V vs. RHE, since at this potential, all catalysts studied show appreciable OER activity, allowing a fair comparison of their charge transfer resistances [43]. Figure 3b shows that the Ni–P_24_ sample deposited for 3 min presented the lowest charge transfer resistance (R_ct_), whose value was 40 ohms. The electrocatalytic kinetics involved in the OER of Ni–P alloys and the Ni substrate were further examined by corresponding Tafel plots, as reported in Figure 3c. The Tafel slope of Ni–P_24_ deposited for 3 min was about 70 mV dec^−1^, which exceeds that of other Ni–P coatings and the nickel substrate, indicating that the oxygen evolution rate was more efficiently promoted for the thinnest Ni–P catalyst. According to an overall electrochemical property examination, the Ni–P catalysts showed a considerable dependence of their OER activity on the thickness, whose reduction positively influenced the performance. Similar results were found for samples with different phosphorous concentrations in the alloy (Figures S2 and S3 display the electrochemical analyses for samples Ni–P_17_ and Ni–P_21_, respectively). As previously reported [32], the reduced activity metrics for the thicker samples can be attributed to a higher potential drop occurring from the substrate to the top of the sample, leaving a high portion of the film at a potential not sufficient to drive the electrochemical reaction.

To ascertain the influence of the phosphorous concentration on the electrochemical activity, we carried out further OER experiments on the thinnest Ni–P samples with different phosphorous concentrations.

The analysis of the polarization and EIS curves reported in Figure 3d,e revealed that, among these samples, the Ni–P_24_ catalysts delivered a superior catalytic activity for the OER with the lowest required overpotential and R_ct_. In regard to the Tafel slopes values, these were quite comparable among the various samples (see Figure 3f).

The intrinsic catalytic activity of an electrocatalyst can be better evaluated considering the TOF normalized to each active site. There are two approaches to determine the TOF which differ in the method of measuring the catalyst number of active sites, that is, the TOF total metal (TOF_tm_), which establishes the lower limit of the TOF, by assuming that all the catalyst atoms participate in OER electrocatalysis [44], and the TOF_redox_, which involves taking into account only the number of metal cations that are electrochemically active, by integrating the redox waves of catalysts displaying clear redox features in the polarization curves (details included in the ESI) [30,45]. As advocated by other authors [44], adopting the former method for TOF estimation is incorrect and misleading, since the TOFsdetermined assuming that the entire mass of the loaded catalyst is active may differ by several orders of magnitude. It is worth noting that most studies do not include TOF determination in electrochemical investigations. Nevertheless, the turnover frequencies are a useful metric to gain a better understanding of the intrinsic catalytic activity, since they are independent of catalyst loading. Herein, we report the TOF_tm_ and TOF_redox_ values obtained in the 300–400 mV overpotential range, in which the OER is in full operation.

**Figure 4:**
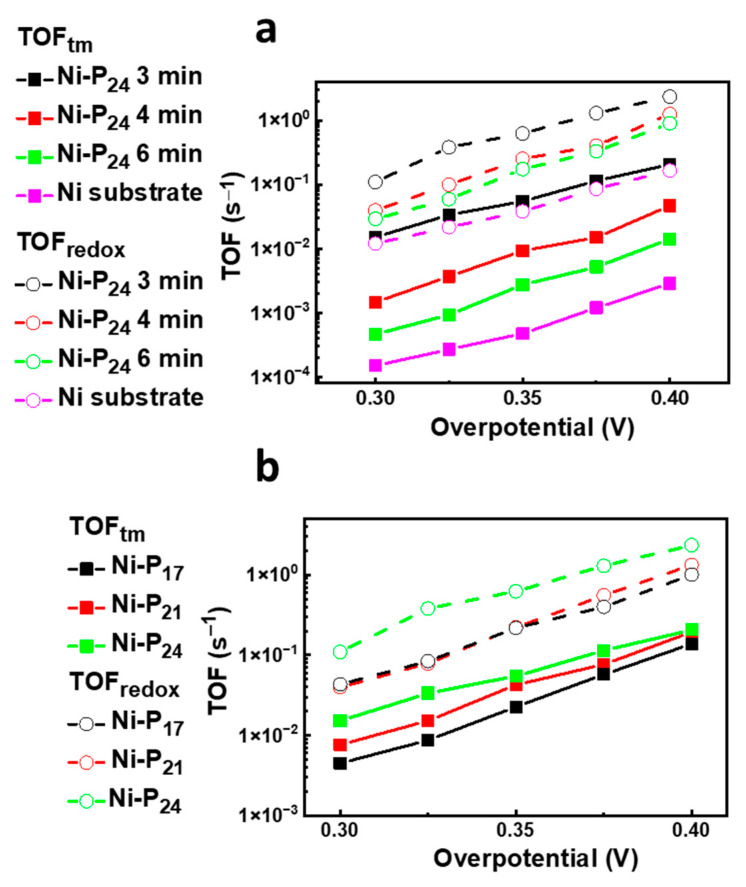
TOF_tm_ and TOF_redox_ of (**a**) Ni–P_24_ samples deposited at different growth times, and (**b**) Ni–P coatings with different percentages of phosphorus deposited for 3 min.

Figure 4 summarizes the trends of the TOF_tm_ and TOF_redox_ values for the Ni–P alloys grown at different deposition times and with different phosphorus concentrations. Clearly, in all cases, the TOF increases with the applied overpotential for kinetics reasons. Remarkably, the spread between the TOF_tm_ and TOF_redox_ values becomes more pronounced by increasing the deposition time; this again emphasizes that the more reliable TOF_redox_ method, where the surface active metal sites are calculated from the redox active peak current, should be used.

Significantly, the TOF values obtained for the Ni–P_24_ catalyst deposited for 3 min generally outperformed those of Ni–P_24_ catalysts deposited for longer amounts of time at each specific overpotential (Figure 4a), revealing that the thinnest catalyst displayed the fastest rate of O_2_ production, thus demonstrating the highest Faradaic efficiency among all samples. Similar results were found for Ni–P_17_ and Ni–P_21_ catalysts (Figure S4a,b). The TOF values determined for the Ni–P_17_, Ni–P_21_ and Ni–P_24_ samples, reported in Figure 4b, point to a higher intrinsic catalytic activity of the Ni–P_24_ catalysts. Notably, the Ni–P_24_ catalysts show TOF_tm_ values that are quite comparable with those presented in the literature, while the TOF_redox_ values overmatch those of other reported Ni–P-based electrocatalysts (see Table 2).

The remarkable TOF values found for this electrode are even more meaningful, indicating an efficient utilization of the electroactive sites for oxygen production.

The superior activity of the sample with a major phosphorous concentration deserves a comment. Compared to unalloyed Ni and P metals, in Ni–P catalysts, the Ni atoms are more positively charged (Ni^δ+^), while the P atoms are more negatively charged (P^δ−^). The partially charged nature of both elements in nickel phosphide allows a more efficient charge transfer between Ni and P, which is further enhanced as the value of δ becomes higher [46]. Furthermore, as reported by J. Li et al. [47], the lower the Ni/P atomic ratio, the higher the average valence state (δ^+^) of the Ni metal active sites in Ni–P catalysts; this can explain the superior intrinsic catalytic activity of Ni–P_24_ among the present Ni–P catalysts.

Summarizing all the electrochemical results hitherto reported, our Ni–P_24_ sample deposited for 3 min ensures the highest intrinsic electrocatalytic OER performance. Indeed, this electrocatalyst showed the best catalytic activity due to its low values of overpotential, charge transfer resistance and Tafel slope and its high TOF.

**Figure 5:**
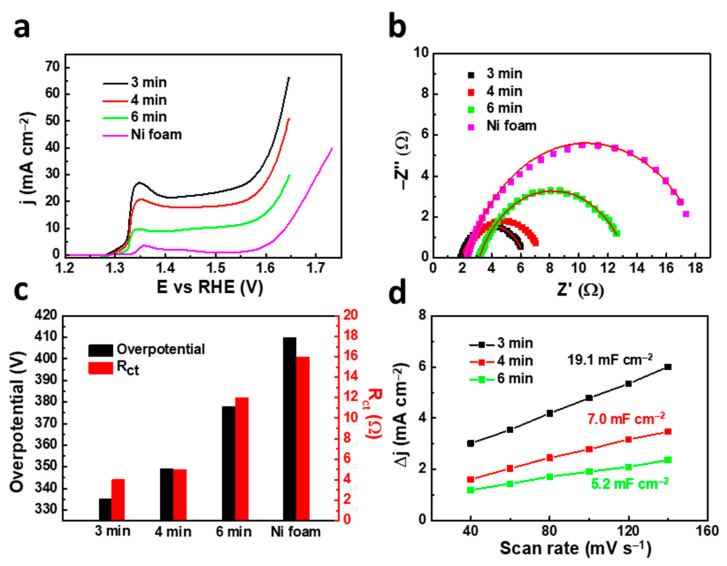
(**a**) Polarization curves of Ni foam and Ni–P_24_ coatings deposited on Ni foam at different times; (**b**) Nyquist plots of the four samples with the fitting curves plotted; (**c**) comparison of overpotential and charge transfer resistance among the four samples; (**d**) the difference between the anodic and cathodic current densities (ΔJ = −J_a_ − J_c_) plotted against the scan rate for catalysts deposited at different times.

An effective approach to optimize the performance of electrocatalysts for water splitting is to deposit them on an electrode with a highly porous structure. In this regard, the use of Ni foam as the electrode endows electrocatalysts with a larger specific surface area and more active sites [17]. Prompted by the interesting results observed in the Ni–P samples deposited on the nickel flat electrode, we decided to deepen our investigation on the nickel foam electrode, in order to evidence how the appropriate nanoporous structure of the electrode may affect the electrochemical performance. The depositions on nickel foam were carried out adopting the same experimental conditions as those used for the Ni–P_24_ coating, which was demonstrated to be the most performant Ni–P alloy. As can be seen in Figure 5a, the polarization curves indicate that the deposition of Ni–P_24_ onto nickel foam enhanced its electrocatalytic performance, since a lower overpotential of 335 mV at a current density of 10 mA cm^−2^ was attained. Remarkably, this value is comparable or even superior to that of other Ni–P electrocatalysts in alkaline media (see Table 2). Concerning the samples deposited for longer times, the results confirm the trend of an overpotential increase with the deposition time. Meanwhile, the OER activity of the bare Ni foam is faint, confirming the pivotal role of the Ni–P coating in the electrochemical performance enhancement. The superior charge transfer capability of the nickel foam electrode compared to the Ni flat electrode was confirmed by EIS measurements (Figure 5b), showing an R_ct_ value that reaches a very low value of only 4 ohms for the thinnest sample. An overview of the overpotential and R_ct_ values found for the four samples is presented in Figure 5c.

Both the lower overpotential and R_ct_ values measured for the samples grown on nickel foam clearly suggest that the Ni–P catalyst deposited on this highly porous electrode can expose and utilize more electroactive sites to boost the electrocatalytic activity.

To thoroughly understand the reasons for the improvement in OER performances upon reducing the catalyst size, we calculated the electrochemical surface area (ECSA) of the catalysts, which is correlated with the surface capacitance. The electrochemical double layer capacitance (C_dl_) was determined by plotting the capacitive current density, calculated from the cyclic voltammogram (CV) curves (Figure S5 and details included in ESI), against the scan rate (Figure 5d).

The C_dl_ of Ni–P deposited for 3 min (14.5 mF cm^−2^) was higher than that of samples deposited for 4 (8.0 mF cm^−2^) and 6 min (5.5 mF cm^−2^). According to the formula ECSA = C_dl_/C_S_, the calculated ECSA was 362.5, 200.0 and 137.5 cm^2^ for the Ni–P catalysts deposited for 3, 4 and 6 min, respectively, revealing that the enhanced electrocatalytic OER activity of the thinnest catalysts is associated with the higher ECSA, which bestows more effective active sites on their surface.

Finally, the durability of the Ni–P/nickel foam electrode was tested using chronopotentiometry. As displayed in Figure S6, the voltage at a current density of 10 mA cm^−2^ was nearly constant upon 12 h of testing, demonstrating the very good stability of the electrode.

## 4. Conclusions

In summary, we used a feasible electroless plating method to develop high-efficiency Ni–P OER catalysts. The comprehensive study, which included properly tuning the plating parameters, allowed for size and compositional control of the deposited alloys. By reducing the thickness, the activity of the catalysts was greatly improved. Moreover, tuning the hypophosphite concentration in the plating bath enabled control of the Ni–P composition in the alloys. The electrochemical measurements showed that the higher the phosphorus content in the alloy, the higher the OER catalytic activity. The Ni–P alloys with optimized size and composition grown on nickel foam substrates required only 335 mV to deliver a current density of 10 mA cm^−2^ for the OER under alkaline conditions, where the charge transfer resistance was as low as 4 ohms, and the TOF had a high value of 0.62 s^−1^. As key factors, the high activity, good stability and low price of the present Ni–P alloys realized by a facile electroless deposition method open the route towards large-scale and long-term applications of metal phosphide OER catalysts, rendering them compelling candidates for application in commercial electrolyzers.

## Figures and Tables

**Table 1:** Composition of various nanostructured Ni–P electrocatalysts realized by tuning the hypophosphite concentration.

Component	Ni–P_17_	Ni–P_21_	Ni–P_24_
NaH_2_PO_2_·H_2_O (g L^−1^)	7.5	15	30

**Table 2:** Activity metrics of Ni–P-based electrocatalysts for the OER.

Catalyst Material	Electrolyte	Overpotential at 10 mA cm^−2^ (mV)	Tafel Slope (mV dec^−1^)	TOF_tm_ (s^−1^)	TOF_redox_ (s^−1^)	Electrode	Reference
NiP nanoparticles	1 M KOH	380	106			GCE	[48]
NiP nanosheets	1 M KOH	230	68		0.0058 (350 mV)	Carbon fiber	[49]
Ni_2_P superstructures	1 M KOH	200	72		0.015 (350 mV)	Ni foam	[50]
Ni_2_P microarrays	1 M KOH	305	110			Ni foam	[51]
Ni_2_P nanowires	1 M KOH	400	60			FTO	[52]
NiP nanocrystals	1 M KOH	260	62	0.074 (300 mV)		GCE	[53]
NiP nanocatalysts	1 M KOH	330	92			GCE	[54]
NiP films	1 M KOH	344	49			Copper foil	[55]
Ni_2_P nanoparticles	1 M KOH	310	71	0.05 (400 mV)		Carbon paper	[22]
Ni_2_P nanoparticles	1 M KOH	350	72			GCE	[56]
NiP spheres	1 M KOH	307	107		0.16 (350 mV)	Carbon paper	[57]
Ni_2_P nanowires	1 M KOH	290	47	0.012 (300 mV)		GCE	[58]
Ni_2_P nanosheets	1 M KOH	N.A.	78			Ni foam	[59]
**NiP nanocoatings**	**1 M KOH**	**388**	**70**	**0.01** **(300 mV)** **0.05** **(350 mV)**	**0.11** **(300 mV)** **0.62** **(350 mV)**	**Ni flat**	**This work**
**NiP nanocoatings**	**1 M KOH**	**335**	**71**			**Ni foam**	**This work**

## Data Availability

The data presented in this study are available in supplementary material.

## Supplementary Materials

The following are available online at https://www.mdpi.com/article/10.3390/nano11113010/s1, Figure S1: SEM micrograph after the OER of Ni–P_24_ coating deposited for 6 minutes, Figure S2: (a) Polarization curves, (b) Nyquist plots with the fitting curves plotted and (c) Tafel plots of Ni–P_17_ coatings deposited at different times, Figure S3: (a) Polarization curves, (b) Nyquist plots with the fitting curves plotted and (c) Tafel plots of Ni–P_21_ coatings deposited at different times, Figure S4. TOF_tm_ and TOF_redox_ of (a) Ni–P_17_ and b) Ni–P_21_ samples deposited at different growth times, Figure S5: Cyclic voltammograms in the region of 0.15–0.25 V vs. Hg/Hg_2_Cl_2_ with different potential scanning rates of Ni–P_24_ coatings deposited on Ni foam for (a) 3, (b) 4 and (c) 6 minutes, Figure S6: Chronopotentiometric response of Ni–P_24_ sample deposited for 3 min on nickel foam held at 10 mA cm^−2^ for 12 h.
