# Supplementary material for: Optimization of Oxygen Evolution Reaction with Electroless Deposited Ni–P Catalytic Nanocoating
Source: Nanomaterials (Basel). 2021 Nov 9;11(11):3010. doi: 10.3390/nano11113010 (PMC8623144; doi:10.3390/nano11113010)
Supplement: Supplementary file 1 [file nanomaterials-11-03010-s001.zip › nanomaterials-1432627-supplementary.pdf]

nanomaterials

Supporting Information

Optimization of Oxygen Evolution Reaction with Electroless Deposited Ni–P Catalytic Nanocoating

Sergio Battiato [1,*], Mario Urso [1], Salvatore Cosentino [1], Anna Lucia Pellegrino [2], Salvo Mirabella [1] and Antonio Terrasi [1]

[1] IMM-CNR and Università di Catania, Via S. Sofia 64, 95123 Catania, Italy; mario.urso@ct.infn.it (M.U.); salvatore.cosentino@ct.infn.it (S.C.); salvo.mirabella@dfa.unict.it (S.M.); antonio.terrasi@ct.infn.it (A.T.)
[2] Dipartimento di Scienze Chimiche, Università degli Studi di Catania, INSTM UdR Catania, Viale Andrea Doria 6, I-95125 Catania, Italy; annalucia.pellegrino@unict.it
* Correspondence: sergio.battiato@dfa.unict.it

TOF calculation

The TOF is defined as the rate of production of oxygen molecules per available (and active) site:

$$TOF = j/4nF$$

where j is the measured current density at a certain overpotential, F is the Faraday constant (96 485 C mol^{-1}) and n is the number of moles x cm^{-2} of the available (active) sites determined considering all the total metal as active (TOF_{tm}) or the amount of electrocatalysts participating in the OER by analysing the oxidation peak in the CV curves by the redox peak integration (TOF_{redox}).

Calculation of n for TOF_{tm}

Avogadro's number – 6.022×10^{23} at/mol
Atomic density of Ni-P (at/cm^2) - determined by RBS spectra

$$n\ (TOF_{tm}) = \frac{\text{Atomic density of Ni-P(at/cm}^2\text{)}}{\text{Avogadro's number (at/ mol)}} = \text{n of Ni-P (mol/cm}^2\text{)}$$

Calculation of n for TOF_{redox}

The amount of charge passed through the electrode during the redox reaction can be derived by integrating the redox peak in the cyclic voltammetry.

Thus, the n for TOF_{redox} can be calculated by the following expression:

$$n\ (TOF_{redox}) = \frac{\text{Q calculated from redox peak (C)}}{\text{Faraday constant}\left(96485\ \frac{\text{C}}{\text{mol}}\right) \times \text{testing area of he electrode (cm}^2\text{)}} = \text{n of Ni-P (mol/cm}^2\text{)}$$

Calculation of C_{dl} and ECSA

The double layer capacitance (C_{dl}) was evaluated by cyclic voltammetry in non-Faradaic potential range 0.15–0.25 V vs Hg/Hg_2Cl_2 at various scan rates (40, 60, 80, 100, 120, 140 mV s^{-1}). Plotting the capacitive current density ($|j_a - j_c|$) determined at 0.20 V vs. the scan rate (υ) yielded a straight line with a slope equal to twice the C_{dl}. The electrochemical active surface area is then calculated from the electrochemical double layer capacitance according to the expression [1]: ECSA = C_{dl}/C_s , where C_s is the specific capacitance of the material or the capacitance of an atomically smooth planar surface of the material per unit area under identical electrolyte conditions. Here, we adopt a general specific capacitance of 0.04 mF cm^{-2}, typical value reported for a metal electrode in 1.0 M KOH solution [2].

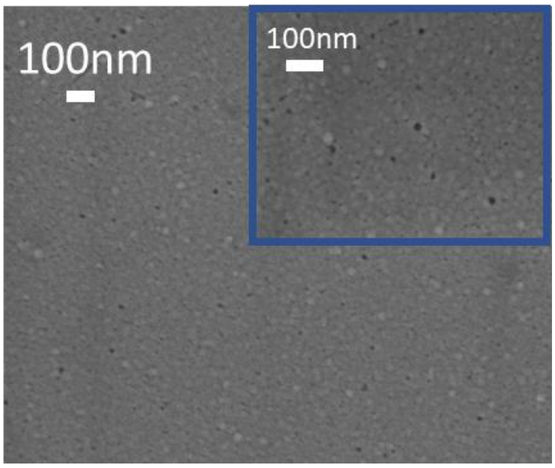

Figure S1. SEM micrograph after OER of Ni-P_{24} coating deposited for 6 minutes.

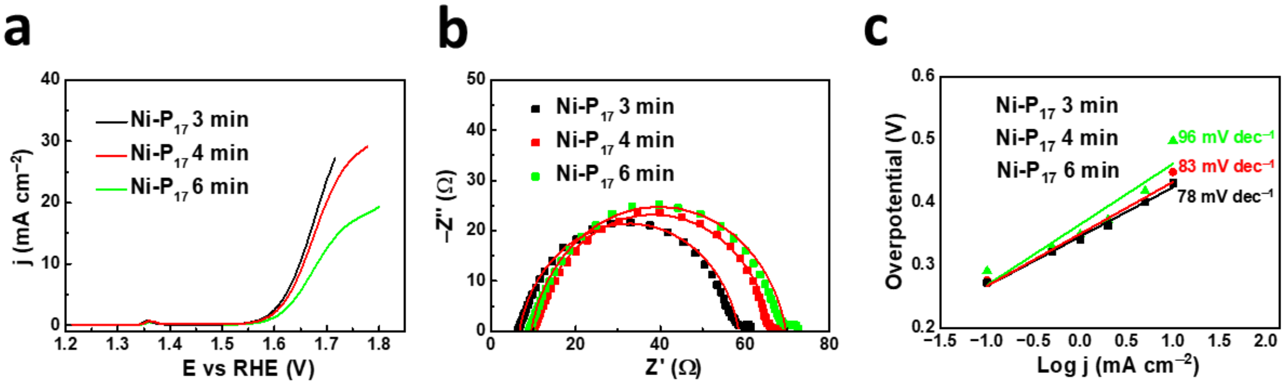

Figure S2. (**a**) Polarization curves, (**b**) Nyquist plots with the fitting curves plotted and (**c**) Tafel plots of Ni-P_{17} coatings deposited at different times.

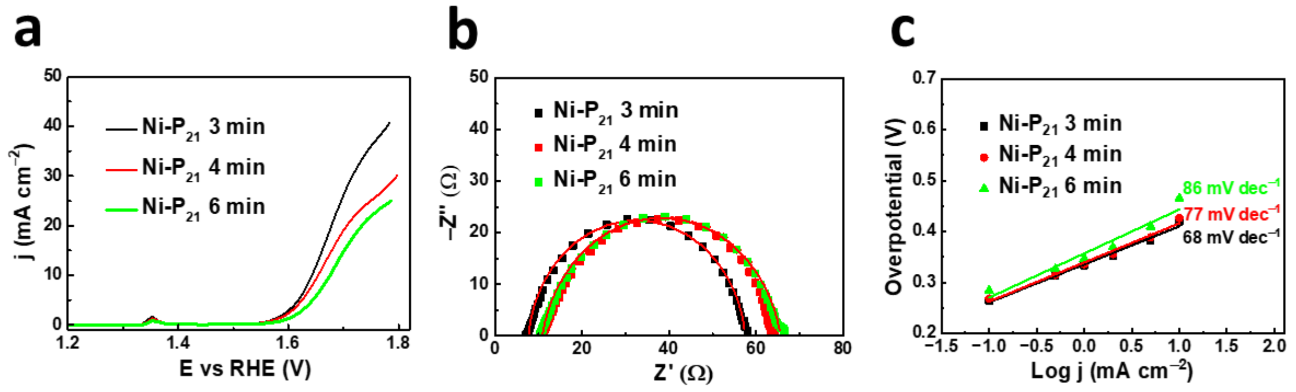

Figure S3. (**a**) Polarization curves, (**b**) Nyquist plots with the fitting curves plotted and (**c**) Tafel plots of Ni-P_{21} coatings deposited at different times.

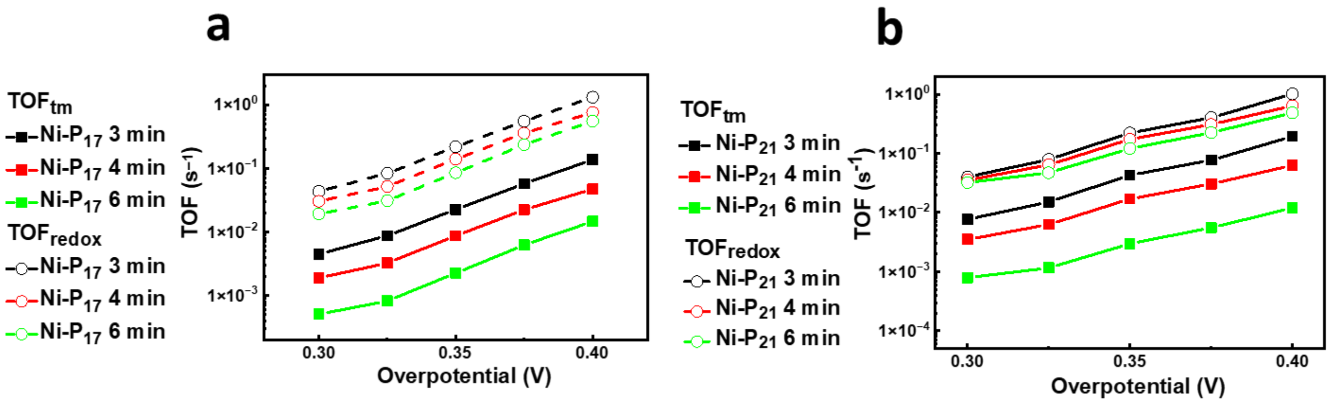

Figure S4. TOF_{tm} and TOF_{redox} of (**a**) Ni-P_{17} and (**b**) Ni-P_{21} samples deposited at different growth times.

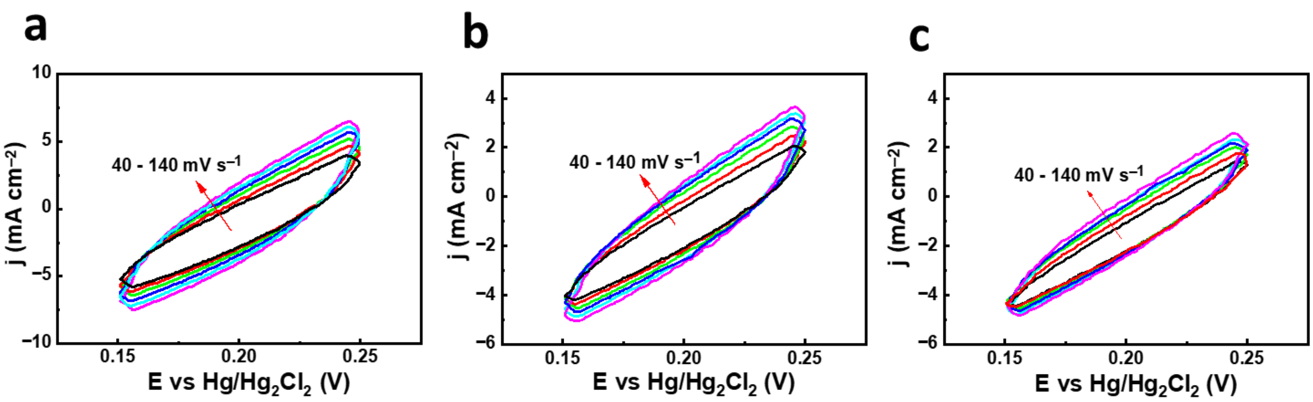

Figure S5. Cyclic voltammograms in the region of 0.15-0.25 V vs. Hg/Hg_2Cl_2 with different potential scanning rates of Ni-P_{24} coatings deposited on Ni foam for (**a**) 3, (**b**) 4 and (**c**) 6 minutes.

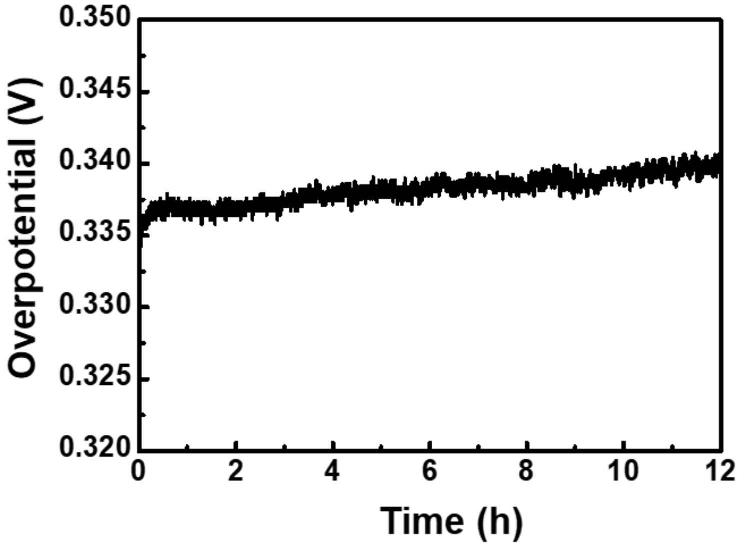

Figure S6. Chronopotentiometric response of Ni-P_{24} sample deposited for 3 minutes on nickel foam held at 10 mA cm^{-2} for 12h.

References

1. McCrory, C.C.L.; Jung, S.; Ferrer, I.M.; Chatman, S.M.; Peters, J.C.; Jaramillo, T.F. Benchmarking hydrogen evolving reaction and oxygen evolving reaction electrocatalysts for solar water splitting devices. *J. Am. Chem. Soc.* **2015**, 137, 4347–4357.
2. Ahmed, A.T.A.; Hou, B.; Chavan, H.S.; Jo, Y.; Cho, S.; Kim, J.; Pawar, S.M.; Cha, S.; Inamdar, A.I.; Kim, H.; Im, H. Self-assembled nanostructured CuCo2O4 for electrochemical energy storage and the oxygen evolution reaction via morphology engineering. *Small* **2018**, 14, 1800742.
